# The role of extracellular vesicles in osteoarthritis treatment via microenvironment regulation

**DOI:** 10.1186/s40824-022-00300-7

**Published:** 2022-10-05

**Authors:** Han Yin, Muzhe Li, Guangzhao Tian, Yang Ma, Chao Ning, Zineng Yan, Jiang Wu, Qian Ge, Xiang Sui, Shuyun Liu, Jinxuan Zheng, Weimin Guo, Quanyi Guo

**Affiliations:** 1 Institute of Orthopedics, The First Medical Center, Chinese PLA General Hospital, Beijing Key Lab of Regenerative Medicine in Orthopedics, Key Laboratory of Musculoskeletal Trauma and War Injuries PLA, No. 28 Fuxing Road, Haidian District, Beijing, 100853 PR China; 2 Department of Orthopedics, The First Affiliated Hospital of University of South China, Hengyang, 421000 China; 3 School of Medicine, Nankai University, Tianjin, 300071 China; 4 Huaiyin People’s Hospital of Huai’an, Huai’an, 223001 China; 5 Department of Orthodontics, Guanghua School of Stomatology, Guangdong Provincial Key Laboratory of Stomatology, Sun Yat-Sen University, No.56 Linyuan Xi Road, Yuexiu District, Guangzhou, Guangdong 510055 People’s Republic of China; 6 Department of Orthopaedic Surgery, Guangdong Provincial Key Laboratory of Orthopedics and Traumatology, First Affiliated Hospital, Sun Yat-Sen University, No.58 Zhongshan Second Road, Yuexiu District, Guangzhou, 510080 Guangdong China

**Keywords:** Extracellular vesicles, Nanomaterials, Osteoarthritis, Microenvironment, microRNAs

## Abstract

Osteoarthritis (OA) is a degenerative joint disease that is common among the middle-aged and older populations, causes patients to experience recurrent pain in their joints and negatively affects their quality of life. Currently, therapeutic options for patients with OA consist of medications to alleviate pain and treat the symptoms; however, due to typically poor outcomes, patients with advanced OA are unlikely to avoid joint replacement. In recent years, several studies have linked disrupted homeostasis of the joint cavity microenvironment to the development of OA. Recently, extracellular vesicles (EVs) have received increasing attention in the field of OA. EVs are natural nano-microcarrier materials with unique biological activity that are produced by cells through paracrine action. They are composed of lipid bilayers that contain physiologically active molecules, such as nucleic acids and proteins. Moreover, EVs may participate in local and distal intercellular and intracellular communication. EVs have also recently been shown to influence OA development by regulating biochemical factors in the OA microenvironmental. In this article, we first describe the microenvironment of OA. Then, we provide an overview of EVs, summarize the main types used for the treatment of OA, and describe their mechanisms. Next, we review clinical studies using EVs for OA treatment. Finally, the specific mechanism underlying the application of miRNA-enriched EVs in OA therapy is described.

## Introduction

OA is a degenerative joint disease that occurs in the knee joint. OA involves structural changes in hyaline cartilage, subchondral bone, ligaments, the joint capsule, the synovial membrane, and periarticular muscles. Local damage to the articular cartilage centered on weight-bearing areas is a representative pathological feature of OA. Worldwide, 9.6% of men and 18.0% of women over 60 years of age experience OA symptoms. Approximately 80 percent of people with OA have substantially reduced mobility, and 25 percent are unable to perform major daily tasks [1]. Currently, a gold standard therapy is unavailable for OA, although surgery and medications have achieved some success in treating patients. However, the safety and risks associated with surgery, as well as the side effects of drugs, remain clinical issues. An understanding of the pathogenic processes that drive OA might provide opportunities for future development of therapies to address this unmet clinical need.

OA is a low-grade inflammatory disease that causes cartilage degradation, synovial inflammation, subchondral bone alterations, osteophyte development, ligament degeneration, joint capsule hypertrophy, and proangiogenic characteristics [2, 3]. Synovitis refers to inflammatory alterations to the synovium, such as hyperplasia of the synovial lining, inflammatory cell infiltration, neoangiogenesis, and fibrosis [4–6]. Synovitis affects 70% of OA patients, and the severity of this condition is related to pain and cartilage loss [7, 8]. Synovial tissue from early OA patients shows elevated production of proinflammatory mediators, suggesting that acute synovitis is one of the first joint alterations to occur [9].

Many processes and substances, including transcription factors, epigenetic changes, cytokines, and proteases, govern joint tissue homeostasis, which is disturbed with OA [6]. This disturbance produces widespread alterations and prevents the synovial joint from facilitating frictionless and smooth mobility. Inflammation and thickening of synovial tissue are caused by this disruption [10], but proinflammatory mediators produced by OA immune cells from the synovium and infrapatellar fat pad (IPFP) also contribute to cartilage destruction [11]. Pattern recognition receptors such as Toll-like receptors recognize distinct pathogen-associated molecular patterns and damage-associated molecular patterns [including extracellular matrix (ECM) degeneration and products of cellular stress], prompting the cells present in the OA joint to release large amounts of inflammatory mediators. Activation of pattern recognition receptors induces cell signaling, resulting in the production of proinflammatory cytokines and chemokines such as interleukin (IL)-6, IL-8, IL-1, and tumor necrosis factor-α (TNF-α), as well as proteases such as matrix metalloproteinases (MMP)-1, MMP-3, and MMP-13, that degrade the structural components of cartilage ECM (primarily aggrecan (ACAN) and collagen) and alter chondrocyte viability and glycosaminoglycan(GAG)release [6, 11–17]. In patients with OA, large numbers of proinflammatory macrophages (M1) are activated, and these activated M1 macrophages cause further damage to the articular cartilage. Promoting the polarization of M1 macrophages to anti-inflammatory macrophages (M2) is a beneficial approach to protect articular cartilage and promote cartilage regeneration and repair. An imbalance in remodeling mediated by bone resorption by osteoclasts and bone creation by osteoblasts results in a decrease in tissue mineralization, a loss of stiffness, and thickening of the subchondral bone [18].

**Fig. 1:**
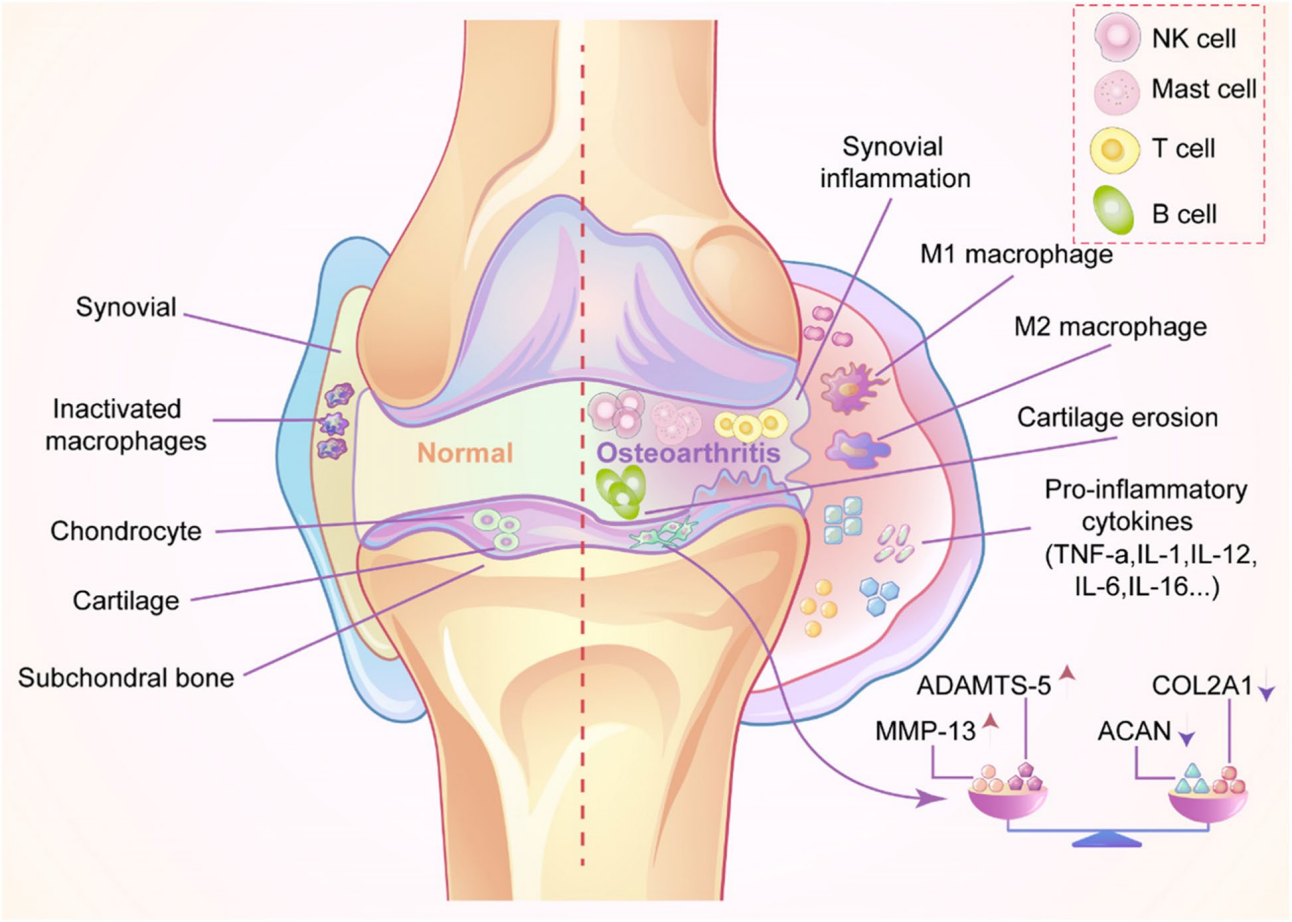
Comparison of the microenvironment between normal and OA joint cavities. Synovial thickening, synovial inflammation, massive activation of inflammatory M1 macrophages, articular cartilage erosion, and the release of a large number of inflammatory factors (including TNF-α, IL-1, IL-12, IL-6, and IL-16), increase in the levels factors responsible for cartilage destruction, and destruction of factors responsible for cartilage formation occur. B cells、T cells、mast cells and NK cells infiltrate into the joint cavity. The imbalance in the homeostasis of the OA joint cavity microenvironment promotes the formation and progression of OA

The abovementioned factors involved in the pathogenesis of OA cause an imbalance in the microenvironmental homeostasis of the joint cavity, which further aggravates the disease. This microenvironment has been summarized as presenting dysregulation of anti-inflammatory and proinflammatory factors, an imbalance in the immune system, disruption of the chondrogenic factor, and an increase in the amount of destructive factors (Fig. 1). Therefore, accurate regulation of the OA microenvironment and restoration of homeostasis are essential to protect articular cartilage and slow the development of OA. EVs and their regulatory potential have been the subject of many recent studies. EVs participate in cell-to-cell communication. They are produced by cells and are considered natural nano-microcarrier materials that, unlike other biological materials, inherit the biological characteristics of their precursors. EVs also have low toxicity and excellent selectivity, as well as the ability to penetrate biological membranes and transport a large number of bioactive molecules between cells. EVs were linked to OA in recent studies, as EVs regulate the inflammatory response and promote M2 macrophage polarization, cartilage production, and tissue healing [19–22]. In addition to attracting interest for other therapeutic applications, EVs may play a role in the treatment of OA by controlling the OA microenvironment. Moreover, the creation of nanomaterials based on EVs or their derivatives will be a new avenue for future cell-free OA biotherapy.

In this review, we first present the microenvironment of OA. Then, we will provide an overview of EVs and the main types of EVs used to treat OA. Finally, we review the main mechanisms of EVs in the treatment of OA.

## The microenvironment of OA

OA is a disease that can affect the cartilage and surrounding tissues of any joint, although it most typically affects major joints, such as the knee, hip, and wrist. According to epidemiological data, OA affects approximately 4% of the global population, with basically no sex or geographic disparities. The annual incidence of OA-related disability accounts for 2% of all disability rates. Men are more likely than women to experience OA before the age of 45 years, while women are more likely to experience OA after the age of 45 years. The incidence increases progressively with age, reaching up to 40% or more in some areas in adults over the age of 60 years, 20% of whom will experience substantial symptoms at a certain stage. With the aging of the population, the incidence of OA will increase, thus increasing the costs of medical treatment. Therefore, to the identification of effective strategies to prevent and cure OA has become increasingly essential. The next section provides an overview of the microenvironmental changes that occur in the joints throughout the pathogenesis of OA.

### Cytokines

The OA microenvironment is incredibly complex, containing many different cells types that release a variety of cytokines. Macrophages, which are categorized into either the M1 or M2 type, are the most important of these cell types. A considerable imbalance in the ratio of M1 to M2 macrophages has been observed in the OA microenvironment, with an increase in the M1 population and a reduction in the M2 population, which contributes to the development of OA.

Cytokines stimulate cells, which in turn activate the local cells (synovial cells, chondrocytes, osteoblasts, osteoclasts, etc.), causing pathological changes such as cartilage degradation, vascular proliferation, and bone production through endocrine, autocrine, and paracrine signaling [23]. IL-1β and TNF-α are the most important of these cytokines. The most potent cytokine for cartilage degradation in the OA microenvironment is IL-1β [24], which is produced by chondrocytes, osteoblasts, synoviocytes, and leukocytes. IL-1β acts independently or synergistically with other cytokines to cause articular cartilage degradation and joint inflammatory responses [25]. According to Piotr Wojdasiewicz et al. [26], IL-1β activates its downstream transcription factors to promote synovial vasodilation, hyperplasia, and joint discomfort, resulting in the expression of hundreds of genes, some of which produce inflammatory mediators, including nitric oxide (NO) and prostaglandin E2 (PGE2). By inducing the generation of interstitial collagenase, matrix lysin 1 and collagenase 3 [27], and platelet-reactive protein motif-containing disintegrin-like metalloproteinases, IL-1β disturbs chondrocytes and the ECM, damaging the cartilage structure and even inducing localized abnormalities in the articular cartilage. These changes further amplify the activity of IL-1β by inducing the production of TNF-α, other cytokines, such as IL-6 and IL-8, chemokine (C–C motif) ligand 5 (CCL5), and different adhesion molecules by chondrocytes, osteoblasts, synoviocytes, and leukocytes in an autocrine manner. IL-1β also induces the generation of reactive oxygen species (ROS) during disease progression [28], which produces different peroxides that directly degrade articular cartilage and exacerbate OA symptoms.

TNF-α is the another inflammatory cytokine that triggers an inflammatory response in people with OA [29]. TNF-α stimulates the release of MMP-1, MMP-3, and MMP-13 from cartilage, the synovium, and subchondral bone layer-associated cells, resulting in a gradual decrease in the cartilage collagen and proteoglycans contents and inhibition of proteoglycan and collagen II (COL II) synthesis. These changes ultimately indirectly leads to chondrocyte death and disturbs the homeostatic balance between cartilage damage and repair, resulting in varying degrees of chondrocyte death and disruption [30]. TNF-α can induces the proliferation of vascular endothelial cells by stimulating tissue expression of growth factors such as vascular endothelial growth factor, basic fibroblast growth factor, and platelet-derived growth factor, which promotes vasodilation and subchondral bone and synovial tissue proliferation and exacerbates the inflammatory symptoms of OA, as noted by Wang et al. [31]. TNF-α may also reduce the activity of the protein kinase CK2 [32]. Furthermore, TNF-α may cause chondrocyte death through both apoptosis and autophagy, worsening cartilage degradation in individuals with OA.

Cytokines have bidirectional functions in tissues such as articular cartilage, the synovium, and subchondral bone. The overexpression of cytokines may lead to the overexpression of downstream molecules such as MMPs, which can contribute to pathological alterations, including cartilage deterioration. As a result, one of the features of the OA microenvironment is the disturbance of the homeostatic equilibrium maintained by cytokines.

### Proteinases

Several proteases that have been implicated in early OA articular cartilage degradation are considered diagnostic and therapeutic criteria for OA. Cellular tissues and immune cells release inflammatory mediators such as IL-1, IL-7, and TNF-α after external activation of the joint [33]. These inflammatory mediators increase the production of several proteases, such as MMPs, a disintegrin and metalloprotease (ADAM), and ADAM with thrombospondin motifs (ADAMTS), which can cause structural damage to articular cartilage and the adjacent joint tissues [34]. The major components of cartilage ECM are ACAN and COL II, which have the primary purposes of maintaining the mechanical structure and biochemical characteristics of articular cartilage.

MMPs are zinc-dependent endonucleases that regulate the composition of the cellular matrix during the normal physiological processes of the organism. MMPs are the most prominent proteases involved in the destruction of cartilage ECM in the OA microenvironment [35]. MMP-13, which is generated by chondrocytes and fibroblasts, is the most effective collagen-degrading enzyme in the MMP family [33]. Furthermore, MMP-13 is a highly active protease involved in the loss of cartilage ECM in the OA microenvironment due to its dual effects on degrading both proteoglycans and the ECM [36, 37]. As a result, the OA microenvironment overexpresses the MMP family of enzymes.

Similar to MMPs, the ADAMTS family of proteases are zinc metalloproteases with platelet-responsive protein motifs. ADAMTS gene expression contributes to the organism homeostasis under normal conditions. However, in the OA microenvironment, inflammatory cytokines such as IL-1 and TNF-α cause a significant increase in the levels of ADAMTS family members, which leads to the development of nonclassical OA inflammation via a downstream chain reaction [38]. Early cartilage deterioration in OA is assumed to involve the hydrolysis of cartilage ACAN by ADAMTS. According to the literature, ADAMTS-5, a member of the ADAMTS family of proteases, is the main hydrolase responsible for the degradation of ACAN in the ECM of OA articular cartilage [39]. Because ADAMTS is involved in total tissue regeneration, increased ADAMTS expression may be linked to not only the degeneration and deterioration of the joint but also the persistence of OA inflammation and injury, implying that OA is an uncontrolled healing process [40].

### Immune cell infiltration

According to Liu et al. [41], the incidence and progression of OA may be associated with the increased infiltration of memory B cells, mast cells, and macrophages and the reduced infiltration of memory CD4 T cells and activated NK cells. By releasing inflammatory mediators and antibodies, B cells control ECM degradation [42, 43]. Mast cell-derived trypsin, according to Wang et al., causes inflammation, chondrocyte death, and cartilage degradation [44]. Proteoglycan degradation has been observed in cocultures of activated mast cells and chondrocytes, according to Woolley et al. [45]. These findings suggest that mast cells causes cartilage deterioration. De Lange-Brokaar, et al. [46] found a significantly greater numbers of mast cells higher in OA samples than in RA’s,and these cells were associated with structural damage in patients with OA, suggesting a role for mast cells in this disease. Macrophages may control the severity of OA and joint inflammation by secreting a variety of mediators.Apparently, regulating the functional phenotype of macrophages may effectively cure OA or promote cartilage repair and regeneration [47, 48]. By secreting cytokines and growth factors, T cells cause the ECM degradation and remodeling [49]. As shown in the study by Ezawa et al.,anincrease in the number of memory CD4 T cells is a common phenomenon observed in the local inflammatory response of OA joints and plays a role in the development of OA [50]. NK cells can modulate the immune system. Based on accumulating evidence,NK cells are key to promoting immune cells involved in OA, and their interaction is facilitated by the CXCL10/CXCR3 axis. NK cells have the ability to alter subchondral bone metabolism and repair in addition to causing cartilage loss [51]. It was discovered that IL-2-activated NK cells may lyse both allogeneic and autologous mesenchymal stem cells [52]. Additionally, NK cells that have been activated can promote osteoclast development [53]. According to the studies reviewed above, OA is caused by the actions of B cells, mast cells, macrophages, T cells, and NK cells. These findings imply that the infiltration of different immune cells, which is a component of the OA microenvironment, is crucial for the pathogenesis of OA.

### Cartilage injury and degeneration

Articular chondrocytes are dormant cells that do not multiply after maturation unless a traumatic or pathogenic event occurs [54]. Cellular degeneration is a natural aging event in which cells stop growing, regress, and lose their ability to proliferate. The lack of neovascularity in articular cartilage inhibits its capacity to regenerate, increasing the difficultly of repair after injury. The increased levels of damaging cytokines and proteases in OA causes articular cartilage erosion, extensive cartilage ECM degradation, and joint surface unevenness. Additionally, the cytokines and proteases mentioned above operate on chondrocytes, altering their biological function and hastening their degeneration and senescence. Degenerated and senescent cells produce important substances in the tissue milieu, which may alter the tissue microenvironment and damage nearby tissues, such as articular cartilage.

## Overview of EVs

### EVs biogenesis

**Fig. 2:**
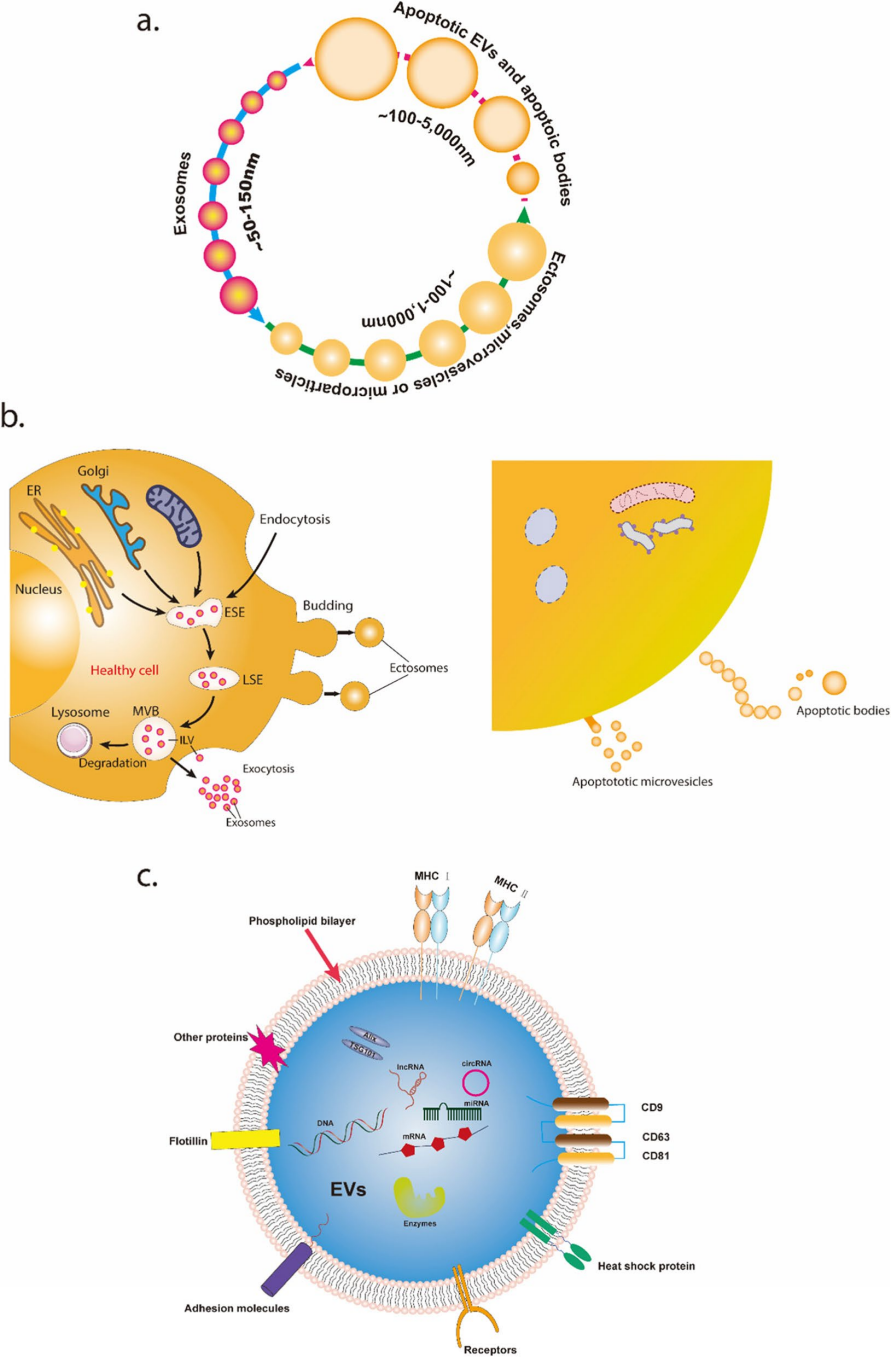
The sizes, contents, and formation mechanisms of the three types of EVs. **a** The size ranges of three types of EVs. **b** EVs biogenesis. **c** The contents of EVs from healthy cells

Pericellular vesicles were initially discovered in mammalian tissues and bodily fluids in the late 1960s [55, 56]. The term "extracellular vesicles" was first used in 2011 to characterize all extracellular structures surrounded by lipid bilayers. The three types of EVs that are classified according to their size, are ectosomes, exosomes (Exos), and apoptotic cell-derived EVs (ApoEVs). The sizes, contents, and formation mechanisms of the three types of extracellular vesicles are all different (Fig. 2). Ectosomes (100–1000 nm in diameter), comprising microvesicles (MVs), microparticles, and large and small vesicles, are very small vesicles that are expelled externally through the plasma membrane. Exos are endosomal vesicles of 50–150 nm in diameter that are produced by repetitive plasma membrane invaginations. Multivesicular bodies (MVBs) containing intraluminal vesicles (ILVs) are formed after the early creation of cup-like structures, early sorted endosomes (ESEs), and late sorted endosomes (LSEs). Both Exos and MVs have the ability to mediate intercellular communication and immunological control.

Ectosomes and Exos are two types of EVs that are generated by healthy cells, although apoptotic cells can also release a variety of EVs. ApoEVs are divided into two types: large membrane-bound vesicles (apoptotic vesicles, 1000–5000 nm) and very small apoptotic MVs (less than 1000 nm). ApoEVs have been proven in several investigations to exhibit activities comparable to those of healthy cell-derived EVs. The primary tasks of ApoEVs include removing apoptotic cells and providing a method of intercellular communication. The therapeutic functions of EVs produced from live cells in the treatment of OA are the topic of this review.

### EVs enrichment

Ultracentrifugation, gradient ultracentrifugation, coprecipitation, size-exclusion chromatography, field flow fractionation, and affinity capture are the methods being used for EVs enrichment and purification. EVs have also been isolated and extracted using techniques such as microporous filtering, microfluidics, and high-performance liquid chromatography.

The prominent approach for used for EVs separation is ultracentrifugation [57]. This method feasibly separates particles with varying settling rates and then eliminates the undesired components during each centrifugation cycle by increasing the centrifugation speed and/or time in a stepwise manner. Although ultracentrifugation is the most widely used technique for EVs isolation, it has several drawbacks, including bulkiness, a requirement for expensive instrumentation, being time-consuming, carrying the risk of contamination with particles of aggregated proteins and ribonucleoproteins, and a requirement for large amounts of sample. Gradient ultracentrifugation, size-exclusion chromatography, and field flow fractionation all face difficulties when separating particles based on particle density or size. In contrast to these physical-based isolation approaches, coprecipitation is a polymer coprecipitation-based EVs enrichment technology. Typically, this approach decreases the hydration of EVs, resulting in their precipitation. A low centrifugal force enables simple and repeatable separation of precipitated EVs products, eliminating the need for a time-consuming ultracentrifugation process [58, 59]. However, this technique is costly and lacks specificity for EVs. Multiphase polymer particles, as well as coprecipitated lipoproteins and argonaut-2 (Ago-2) RNA complexes, are common byproducts of this method. Using phase interactions between markers on the EVs surface and captured molecules linked to distinct carriers, affinity capture enables the extraction of EVs with greater purity but lower yield [60].

Each approach has benefits and drawbacks, and a combination of methods may be the best option to extract the separated EVs. The features of several EVs separation techniques are summarized in Table 1.

**Table 1:** Overview of EVs enrichment methods

Enrichment method	Principle	Advantages	Limitations	Reference(s)
Ultracentrifugation	Density	The most commonly used and well-established program Simple Relatively high yield	Bulky Requires expensive instruments Time-consuming Contamination with aggregated protein and ribonucleoprotein particles Requires a large amount of sample Low purity	[61–64]
Gradient ultracentrifugation	Based on the density gradient of the solution	Most commonly used method Relatively high purity Maintains EV integrity	Time-consuming Requires a large amount of sample Require expensive instrumentation Lower yield	[61–64]
Size-exclusion chromatography	Particle size and molecular mass	Economic Relatively high purity Maintains EV integrity Multiple eluents	Time-consuming Lack of specificity Difficult to produce on a large scale Contamination	[63]
Field flow fractionation	Particle size and molecular mass	High yield High purity Time-efficient	Lack of specificity Difficult to produce on a large scale Requires complex equipment Difficult to perform	[62, 65]
Coprecipitation	Surface charge	Processing that is easy to use	Lack of specificity Difficult to produce on a large scale	[66]
Affinity capture	Based on the interaction between captured molecules and EVs antigens	High purity Specific separation	High cost Only specific target proteins can be isolated Low yield	[61, 64]

### EVs characterization

Isolated EVs must be properly characterized according to the International Society for Extracellular Vesicles (ISEV) minimal standard report for EVs characterization. Complete EVs characterization encompasses an assessment of both general and specific vesicle characteristics. The surface protein indicators of EVs are often characterized using Western blotting or enzyme-linked immunosorbent assay (ELISA). At least three positive and one negative EVs protein indicators should be described according to the ISEV. Moreover, at least one transmembrane/lipid binding protein (e.g., CD63 and CD9) and one cytoplasmic protein (e.g., TSG101 and ALIX) should be positive protein markers. Imaging methods and biophysical characterization are needed to characterize single vesicles. However, the only imaging techniques capable of acquiring high-resolution EVs images are electron microscopy (EM) and atomic force microscopy (AFM), which are methods that include transmission electron microscopy, scanning electron microscopy, and cryo-electron microscopy. Immunogold electron microscopy is commonly utilized to visualize the staining of certain EVs markers. Nanoparticle tracking analysis (NTA), tunable resistive pulse sensing (TRPS), dynamic light scattering (DLS), and flow cytometry (FC) are all examples of biophysical characterization techniques [67].

A light microscopic single EVs analysis approach (SEA) that enables a reliable assessment of numerous protein biomarkers in a single vesicle was described in a recent study [68]. With this method, EVs are immobilized in a microfluidic chamber, immunostained, and photographed. When vesicles are immobilized on the chip surface, the signal-to-noise ratio is generally greater than that when the vesicles are free-floating or flowing in solution. However, the chemical composition of each EVs and how EVs subpopulations are characterized are still unknown [69].

## Main EVs types used to treat OA

### Natural EVs used in OA therapy

Natural EVs are generally formed from unmodified mesenchymal stem cells (MSCs), which not only have the same biological functions as MSCs but also possess features that MSCs lack. As a result, EVs (including MVs and Exos) have been recommended as a replacement for standard cell-based OA treatments due to the following benefits: (1) a small size, (2) improved safety and fewer side effects due to their natural lipid and surface protein composition [70], (3) lower immunogenicity [70], and (4) protection from degradation and therapeutic site targeting of the therapeutic substances (nucleic acids and proteins) [71, 72], (5) the capacity to overcome various biological hurdles that MSCs are unable to overcome [72], (6) elimination of the need for cell injection, and (7) easy preservation procedures with fewer ethical concerns. EVs for OA research have been extracted from MSCs generated from a variety of sources, including bone marrow, adipose tissue, umbilical cords, synovial membranes/fluid, embryonic stem cells, and induced pluripotent stem cells [70]. Table 2 summarizes and compares the characteristics, size, safety, efficacy, derivation, dose, and animal models used with synovial mesenchymal stem cell-derived EVs (S-MSC-EVs), adipose-derived mesenchymal stem cell-derived EVs (AD-MSC-EVs), bone marrow mesenchymal stem cell-derived EVs (BM-MSC-EVs), and human umbilical cord mesenchymal stem cell-derived EVs (hUMSC-EVs). To make the article more rational, we also summarize the differences in MSCs isolated from different tissue sources in Table 3.

**Table 2:** Characteristics of synovial stem, adipose stem, bone marrow stem, and umbilical cord stem-derived EVs for OA

MSCs-EV	Derivation	Size(nm)	Characteristic	Safety	Dose	Animal model	Efficacy	Reference
S-MSC-EVs	Human	30–150	Overexpressionof miR-140-5p	Not mentioned	100 μL; 10^11^ EV particles/mL	Rat knee OA model induced by cutting the medial collateral ligament and medial meniscus	Promotes the proliferation and migration of chondrocytes;inhibits the progression of OA	[73]
	Human	50–200	Not mentioned	Not mentioned	Intravenous injection;8µL; 1.0 × 10^10^/mL	Collagenase-inducedknee OA mouse model	Promotes the proliferation and migration of chondrocytes; reduces OA progression	[74]
	Human	100–120	Overexpression of miR-155-5p	Not mentioned	Articular cavity injection;30μL;10^11^EV particles/mL	Mouse OA model induced by cold water stimulation at 4 °C	Promotes chondrocyte proliferation and migration, and ECM secretion, and inhibits apoptosis; reduces OA-related damage; promotes cartilage regeneration	[75]
	Human	50–100	Enrichment of miR-129-5p	Not mentioned	Not mentioned	Not mentioned	Reduces OA chondrocyte damage and ECM degradation	[76]
AD-MSC-EVs	Human	115–316	Not mentioned	Not mentioned	Not mentioned	Not mentioned	Anti-inflammatory and antioxidative stress activities	[77]
	Human	30–150	Enrichment of miR-100-5p	Not mentioned	Articular cavity injection;10 μL; 10^10^particles/mL	Induced mouse OA model generated by the destruction of the medial meniscus	Inhibits chondrocyte apoptosis; balances anabolic and catabolic processes	[78]
	Human	185–373	Not mentioned	Not mentioned	Not mentioned	Not mentioned	Anti-inflammatory; regulates chondrocyte metabolism	[79]
	Human	Average 86.46	Not mentioned	Not mentioned	Articular cavity injection;6µL; 1 × 10^8^ particles	Medial meniscus instability (DMM) mouse OA model	Prevents cartilage degeneration and attenuates OA progression; promotes chondrocyte proliferation and migration; regulates the expression of catabolic and synthetic factors; inhibits macrophage infiltration	[80]
BM-MSC-EVs	Rat	Average 100	Enrichment of miR-135b	Not mentioned	Articular cavity injection;100µL; 1 × 10^11^ MSC-EV particles/mL	Rat knee OA model induced by cutting the medial collateral ligament and medial meniscus	Regulates cell proliferation, apoptosis, and differentiation; promotes OA cartilage repair	[81]
	Rabbit	50–150	Not mentioned	Not mentioned	Not mentioned	Not mentioned	Inhibits chondrocyte apoptosis	[82]
	Mouse	105.4–118.6	Not mentioned	Not mentioned	Articular cavity injection;250 ng/5 µL	Collagenase-induced knee OA mouse model	Protects cartilage and bone from degradation	[83]
	Rat	50–150	Enrichment of miR-216a-5p	Not mentioned	Articular cavity injection;200 µL; 200 μg of total sEV protein	Rat OA model induced by cutting the anterior cruciate ligament and eliminating the medial meniscus	Stops cartilage degeneration and attenuates OA progression; promotes chondrocyte proliferation and migration; inhibits apoptosis	[84]
hUMSC-EVs	Human	50–150	Enrichment of miR-23a-3p	Not mentioned	Articular cavity injection; 10 × 10^8^particles/mL	Rat cartilage defect model	Promotes cartilage regeneration	[85]
	Human	Average 120	Enrichment of lncRNA H19	Not mentioned	Articular cavity injection; 200 µL; 1 mg/mL	Rat cartilage defectmodel	Promotes chondrocyte proliferation, matrix secretion, and apoptosis inhibition	[86]
	Human	Average 120	3D cultivation	Not mentioned	Articular cavity injection; 500 μL; 1 × 10^10^particles/ mL	Rabbit cartilage defect model	Promotes chondrocyte proliferation, matrix secretion, and apoptosis inhibition	[87]

**Table 3:** Comparative analysis of MSCs isolated from different tissues

Cell types	Acquisition invasiveness	Expansion characteristics	Immune phenotype	Immunogenicity	Osteogenic differentiation capacity	Chondrogenic differentiation capacity	Adipogenic differentiation capacity
S-MSCs	Big	Strong	Express CD90、CD44、CD105	Low	Strong	Strong	Relatively strong
AD-MSCs	Relatively small	Relatively weak	Highly express CD49d、CD54	Low	Weak	Weak	Strong
BM-MSCs	Big	Weak	Highly express CD49f、PODXL	High	Weak	Relatively strong	Strong
hUMSCs	Small	Strong	Express CD105、CD44、CD13、CD29	Low	Weak	Relatively strong	Weak

### S-MSC-EVs

S-MSCs were extracted from the synovium surrounding a joint for the first time in 2001 [88]. S-MSCs specifically regenerate cartilage [89] and are presumed to be the most promising cells for stimulating cartilage regeneration. In vitro, S-MSCs exhibit excellent chondrogenic differentiation potential [90–92].An intra-articular injection of S-MSCs substantially improves cartilage regeneration in experimental animal models. These cells have also been utilized to treat joint-related disorders such as OA [93–96]. According to several studies have recently, S-MSCs-EVs can successfully stimulate cartilage regeneration and delay the development of OA [73, 74, 97]. Tao et al. [73] discovered that human S-MSCs-Exosomes (S-MSCs-Exos) expressing wingless/integrated (Wnt) 5a and Wnt5b reduced ECM secretion by activating Yes-associated protein (YAP) via alternate Wnt signaling pathways while increasing chondrocyte proliferation and migration. MiR-140-5p-Exos inhibited this adverse effect by targeting RalA. In vitro, human S-MSC-140-Exos increased articular cartilage proliferation and migration without interfering with ECM secretion. However, in vivo, human S-MSC-140-Exos effectively prevented OA in a rat model. Additionally, S-MSC-Exos increased chondrocyte proliferation and migration while inhibiting apoptosis, but had no effect on ECM production or secretion, according to Wang et al. [75]. Qiu et al. discovered that miR-129-5p expression was downregulated in OA patients and IL-1-induced chondrocytes, but high mobility group protein (HMGB) 1 was substantially upregulated [76]. S-MSC-Exos enriched in miR-129-5p decreased chondrocytes apoptosis, whereas S-MSCs-Exos enhanced both the IL-1-mediated inflammatory response and apoptosis in chondrocytes. Upon further investigation of this process, miR-129-5p was shown to bind the 3' untranslated region (3'UTR) end of HMGB1 and suppresses IL-1-mediated HMGB1 overexpression. Overall, this study revealed that miR-129-5p present in S-MSCs-Exos may prevent IL-1-induced OA by blocking HMGB1 release.

### AD-MSC-EVs

AD-MSCs have been shown to have significant abilities to control cartilage regeneration and inflammation. They are regarded as a good source of cells for the treatment of OA [98–103]. However, the mechanism by which AD-MSCs stimulate cartilage repair is unknown. AD-MSCs control the local microenvironment mainly by secreting paracrine trophic factors, to promote repair and regeneration, reduce cartilage degradation, and enhance joint function [100]. According to Tofino-Vian et al., EVs, including MVs and Exos, mostly mediate the paracrine effects of AD-MSCs on osteoblasts in individuals with OA [77]. Wu et al. [78] examined the function of IPFP MSCs-derived Exos (MSCs-IPFP-Exos)in OA and the underlying processes. MSCs-IPFP generate large number of Exos and that these MSCs-IPFP-Exos exhibit an Exos-like morphology. MSCs-IPFP-Exos have been shown to ameliorate OA in vivo by inhibiting apoptosis. Moreover, MSCs-IPFP-Exos increase matrix secretion and decrease the expression of degradation-related factors. Furthermore, by blocking the mammalian target of the rapamycin (mTOR) pathway, MSCs-IPFP-Exos may substantially increase chondrocyte autophagy. Tofio-Vian et al. [79] isolated and identified MVs from human AD-MSCs (hAD-MSCs). Then, they studied the chondroprotective role of these MVs and discovered that they reduced the production of the inflammatory mediators TNF-α, IL-6, PGE2, and NO in IL-1-stimulated OA chondrocytes. When OA chondrocytes were treated with these MVs, the measured MMP activity and MMP-13 expression were reduced, but the expression of the anti-inflammatory cytokines IL-10 and COL II increased considerably. Woo et al. [80] examined the therapeutic potential of hAD-MSCs-derived small EVs (hAD-MSCs-sEVs) in the treatment of OA and the corresponding mechanism. hAD-MSCs-sEVs not only increased human chondrocyte proliferation and migration but also reduced the expression of MMP-1, MMP-3, MMP-13, and ADAMTS-5 by increasing COL II production in the presence of IL-1. An intra-articular injection of hAD-MSCs-sEVs dramatically slowed the development of OA and prevented cartilage degeneration in rats treated with sodium monoiodoacetate and mice with medial surgical damage.

### BM-MSCs-EVs

EVs derived from BM-MSCs have been shown to affect cell fate, including apoptosis, proliferation, invasion, and migration [104, 105]. Furthermore, BM-MSCs-EVs control many physiological and pathological processes, such as the immune response, osteogenesis, fibrosis, and angiogenesis [106–109]. In several studies, BM-MSCs-EVs were shown to stimulate the regeneration and repair of injured tissues, including cartilage and subchondral bone [107, 110–115]. Wang et al. [81] discovered that miR-135b-Exos suppress the expression of transcription factor SP1 in chondrocytes. MiR-135b-Exos promote chondrocyte proliferation and accelerate OA cartilage repair by negatively regulating Sp1 expression. This study may provide a new direction for OA treatment. Li et al. [116] investigated the effect of BM-MSCs-Exos on the etiology and behavioral symptoms of mice with lumbar facet joint OA (LFJ OA). They used BM-MSCs-Exos to treat mice with LFJ OA and detected changes in aberrant nerve invasion in the cartilage and subchondral bone. They discovered that BM-MSCs-Exos may alleviate pain by removing abnormal calcitonin gene-related peptide (CGRP)-positive nerves and abnormal H-vascular development in LFJ subchondral bone. BM-MSCs-Exos also suppress the expression of anti-tartaric acid phosphatase and activation of the receptor activator of nuclear factor-κB ligand (RANKL)-receptor activator of nuclear factor-κB (RANK)-tumor necrosis factor receptor-associated factor 6 (TRAF6) signaling pathway. In addition, subchondral bone remodeling was increased. Qi et al. [82] demonstrated that BM-MSCs-Exos can promote chondrocyte proliferation and significantly inhibit IL-1-induced chondrocyte apoptosis by inhibiting p38 and ERK1/2 phosphorylation and stimulating the Akt signaling pathway, indicating that BM-MSCs-Exos can effectively maintain chondrocyte viability in an inflammatory environment. Cosenza et al. [83] discovered that BM-MSCs-Exos and BM-MSC-derived microparticles can increase the expression of chondrocyte markers (COL II and ACAN) while suppressing catabolism (MMP-13, ADAMTS-5) and inflammation (iNOS) markers. BM-MSCs-Exos and BM-MSCs-derived microparticles also protect chondrocytes from apoptosis and suppress inflammatory macrophage activation. Rong et al. [84] extracted sEVs after the hypoxic stimulation of BM-MSCs. These sEVs were capable of initiating the fast repair and regeneration of osteochondral defects and reducing the development of OA. sEVs can deliver miR-216a-5p to cartilage cells. MiR-216a-5p-sEVs can downregulate Janus kinase 2 (JAK2), promote chondrocyte proliferation and migration, and inhibit apoptosis. In vitro and in vivo, kartogenin (KGN)-BM-MSCs-sEVs treatment resulted in more effective cartilage repair and matrix production than treatment with KGN [117]. In conclusion, BM-MSCs-EVs are a viable therapeutic approach for OA.

### hUMSC-EVs

hUMSCs have the advantages of a large tissue supply, a high growth capacity, a painless collection technique and excellent biological characteristics. According to previous research, hUMSCs may develop into osteoblasts, chondrocytes, adipocytes, and a variety of other cell types [118–120]. In recent investigations, MSCs-EVs have been shown to stimulate cartilage formation [73, 121, 122]. Hu et al. [85] examined the role and mechanism of hUMSCs-sEVs in cartilage regeneration. They found that hUMSCs-sEVs could enhance chondrocyte and human bone marrow mesenchymal stem cells (hBM-MSCs) migration, proliferation, and differentiation. An miRNA microarray revealed that miR-23a-3p was the most abundant miRNAs expressed in hUMSCs-sEVs. After transferring miR-23a-3p, hUMSCs-sEVs could suppress phosphatase and tensin homolog (PTEN) expression while increasing protein kinase B (Akt) expression, therefore enhancing cartilage regeneration. Yan et al. [86] revealed that hUMSCs-Exos may function as a natural carrier of the long noncoding RNA (lncRNA) H19. The lncRNA H19 can increase chondrocyte proliferation, migration, and matrix secretion and inhibit chondrocyte death and senescence. The corresponding mechanism is that lncRNA H19-Exos compete with miR-29b-3p and upregulate forkhead box O3 (FOXO3) expression in chondrocytes. An intra-articular injection of hUMSCs-Exos substantially enhances the healing of cartilage abnormalities. Furthermore, hUMSCs-Exos derived from three-dimensional (3D) culture were more beneficial for cartilage regeneration than those derived from traditional two-dimensional (2D) culture [87].

### EVs derived from other cells

Sang et al. discovered [123] that hydrogels containing chondrocyte-derived Exos can promote cartilage regeneration and repair by controlling the levels of inflammatory factors in the OA microenvironment and polarizing macrophages. Zheng,et al. [124] found that compared with IL-1β stimulated chondrocytes, Exos from normal chondrocytes can prevent the development of OA by reversing mitochondrial dysfunction and polarizing macrophages to the M2 phenotype. According to Wa et al. [125], M2 macrophage-derived Exos exert a therapeutic effect on rats with knee OA (KOA) by suppressing the PI3K/Akt/mTOR pathway and reducing the inflammatory response and pathological damage to the articular cartilage. Tan et al. [126] found that the lncRNA H19 present in fibroblast-like cell-derived Exos can target the mir-106b-5p /TIMP2 axis, increase OA chondrocyte proliferation and migration, block ECM degradation, and attenuate the development of OA.

### Engineered EVs for OA therapy

**Fig. 3:**
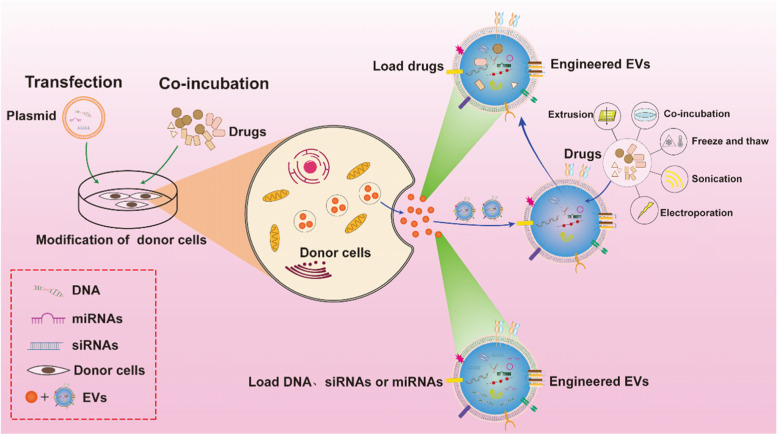
The most important EVs engineering methods. Strategies for designing donor cells are shown on the left. Co-incubation and gene transfection methods are utilized to deliver cargos into donor cells. EVs engineering strategies are shown on the right. Sonication 、electroporation、freeze and thaw、co-incubation and extrusion techniques are utilized to insert cargos into EVs

Engineered EVs have been the focus of scientists in recent years to increase the EVs target specificity and achieve precise control. Engineered EVs outperform natural EVs in terms of therapeutic potential. Engineering procedures (Fig. 3) (e.g., transfection, coincubation, electroporation, sonication, freeze–thaw cycles, extrusion, the use of saponins) are utilized to load EVs with suitable cargo to obtain superior therapeutic effects. Many preclinical experiments analyzing EVs-based medications or molecular delivery have yielded promising results. EVs-encapsulated therapeutic molecules and medications are more stable in the circulation, traverse physiological barriers more easily and have higher biological activity and lower systemic toxicity than their corresponding free molecules. Researchers have devised novel methods to create high-purity and high-yielding EVs and to construct drug or molecule delivery systems with a high loading efficiency, targeting ability, and regulated drug or molecule release to increase the usability of EVs in OA therapy. Liang et al. combined the lysosome-associated membrane protein 2 (LAMP-2B) gene with chondrocyte affinity peptide(CAP) for transfection into dendritic cells to create chondrocyte-targeting EVs and to soften and promote cartilage regeneration. MiR-140 was then transported to chondrocytes deep in the joint [127]. Xu et al. fused the MSC-binding peptide E7 to the EVs membrane protein LAMP-2B to create EVs with E7 peptide (E7-EVs) and synovial fluid mesenchymal stem cell (SF-MSC) targeting capabilities. KGN supplied with E7-EVs enters SF-MSCs more effectively and induces a greater degree of chondrogenic differentiation than KGN provided with EVs alone without E7, indicating that these EVs might be a promising advanced OA stem cell treatment [128].

## Main mechanisms of EVs in the treatment of OA

### Immunomodulation

The principal function of the immune system has long been presumed to be a part of the body's defense mechanism against viruses and the external environment. However, the immune system clearly exerts a significant effect on tissue healing. Proinflammatory cytokines, anti-inflammatory cytokines, and bidirectional factors all exist in the joint cavity, and the dynamic balance between these three variables maintains the normal physiological metabolism of articular cartilage. When this dynamic equilibrium is perturbed, the joint microenvironment is disrupted, which leads to the development of OA. Proinflammatory cytokines such as IL-1, IL-6, and IL-8, as well as MMP-3, are implicated in cartilage injury-induced matrix degradation and joint degeneration [129]. Proinflammatory cytokines, including IL-6 and IL-1 as well as the nuclear factor kappa B (NF-kB) pathway, have been found to exert a significant effect on synovial inflammation and cartilage degradation in OA patients. According to Xia et al. [130],and Zhao et al. [131], EVs produced from AD-MSCs were able to downregulate IL-6 expression and alter the expression of components of the NF-kB pathway.

Synovial and immunological cells, such as macrophages, produce proinflammatory cytokines and MMPs, which contribute to the development of OA [132]. According to recent research, M1 macrophages in OA synovial tissue limit MSC chondrogenic development in vitro via IL-6 [133], and M2 macrophages improve transplant cartilage survival by generating the anti-inflammatory cytokine IL-10 to decrease unfavorable inflammatory responses [134]. As a result, in cartilage regeneration therapy, the proinflammatory milieu of cartilage degeneration or OA must be controlled. Previously, macrophages were divided into two phenotypes: proinflammatory M1 and anti-inflammatory M2 phenotypes. Interferon-γ (IFN-γ), TNF-α, or pathogen-associated molecular patterns activate M1 macrophages. These activated macrophages then release proinflammatory cytokines such as IL-1, IL-6, IL-12, and iNOS. On the other hand, M2 macrophages are activated by different pathways. Transforming growth factor (TGF)-β1 and arginase-1 (Arg-1) are two growth- and angiogenesis-related substances secreted by M2 macrophages that decrease inflammation and promote tissue remodeling [135, 136]. The spatial and temporal distribution of M1 and M2 macrophages is critical to coordinate inflammation and tissue regeneration [137, 138].

**Fig. 4:**
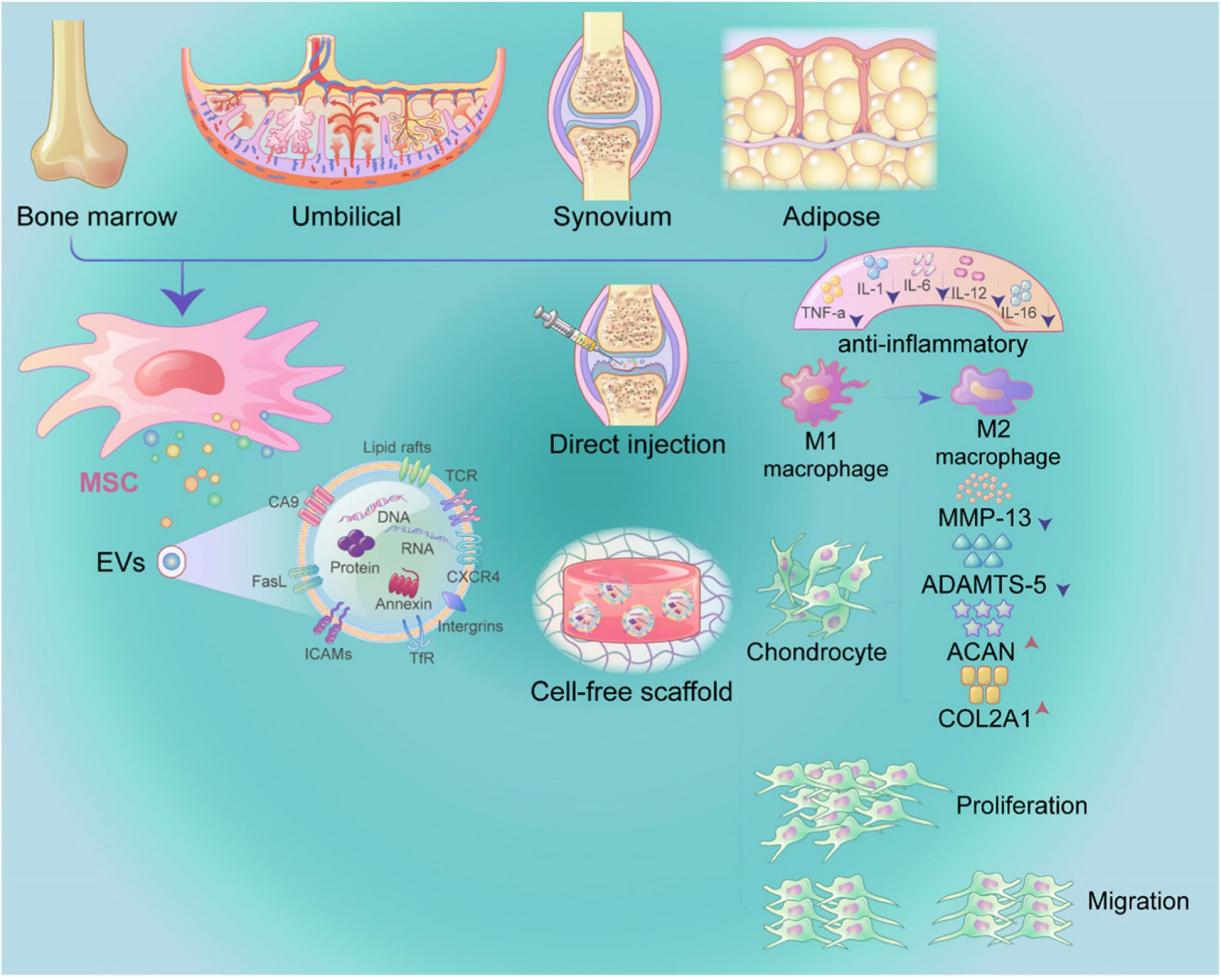
As a new type of natural nanomaterial, EVs secreted by stem cells from various tissues (bone marrow, umbilical cord, synovium, and adipose tissue) regulate the OA microenvironment through various mechanisms to restore the homeostasis of the joint cavity. EVs can be injected directly into the joint cavity in batches or combined with acellular scaffold materials to inhibit inflammatory factor release, and promote the polarization of M1 macrophages to M2 macrophages. Moreover, they also reduce the production of cartilage destruction factors, and promote the synthesis of factors involved in cartilage formation

MSCs-EVs produce large quantities of the anti-inflammatory cytokines IL-10 and TGF-β1 while suppressing the production of the proinflammatory mediators IL-1, IL-6, TNF-α, and IL-12 (Fig. 4). Furthermore, MSC-EVs decrease macrophage activation and promote the M1 to M2 conversion, which is important in many inflammatory illnesses. MSC-EVs exert immunomodulatory effects, according to Zhang et al. They can increase M2 macrophage infiltration into OA cartilage defects and synovial membranes, reduce M1 macrophage infiltration, and downregulate the inflammatory factors IL-1β and TNF-α, resulting in an overall decrease OA inflammatory responses [121]. Although the immunomodulatory effects of EVs on OA are unknown, the presence of EVs in serum was recently shown to protect human OA cartilage from GAG loss in the presence of the inflammatory factor IL-1β. Additionally, the number of M2 macrophages increases following EVs therapy, increasing cartilage regeneration in immunoreactive rats, which has led to our hypothesis that MSCs-EVs might cure OA.

MSCs-EVs also exert anti-inflammatory immunomodulatory effects on T cells, NK cells and B cells. Studies have shown that MSCs-Exo exerts an anti-inflammatory effect on T cells and B cells by reducing the number of CD4 + T cells and CD8 + T cells, increasing the amount of Treg cells, and alleviating the inflammatory response of collagen-induced arthritis [139]. Moreover, MSCs-Exo can promote the transformation of effector T cells into Treg cells and increase the expression level of CTLA-4 in Treg cells. CTLA-4 is an important factor in the immunosuppressive effect of MSCs-Exo [140]. Human dendritic cells (DCs) can release large extracellular vesicles (lEVs), which can effectively induce the activation of CD4 + T cells in vitro. Among EVs secreted by immature DCs, lEVs promote the secretion of Th2 cytokines such as IL-4, IL-5 and IL-13 [141]. In addition, immature dendritic cell-derived exosomes (imDECs) can attenuate the inflammatory response and reduce the infiltration of CD4 + T cells [142]. Tumor-derived exosomes (TDEs) can inhibit the recruitment and migration of NK cells to the tumor environment, while suppressing the secretion of cytokines IFN-γ and TNF-α by NK cells, leading to immune escape and tumor progression [143]. Studies have revealed that MSCs-Exo can inhibit the proliferation of B cells and the differentiation into immunoglobulin-secreting plasma cells, and CCL2 in exosomes directly inhibits the secretion of immunoglobulin antibodies by plasma cells [144]. In conclusion, EVs can exert their anti-inflammatory immunomodulatory effects by regulating T cells, NK cells and B cells. However, there are currently few experimental studies on how EVs regulate T cells, NK cells, and B cells in OA, lacking sufficient basic theory, which is also the focus of the future research.

### Chondrocyte regulation

Inflammation exacerbates the degeneration of damaged/diseased cartilage in OA, resulting in cell death, matrix degradation, and finally a loss of structure and function [129, 132]. Chondrocyte apoptosis is linked to cartilage deterioration and the progression of OA [32], and EVs may help prevent apoptosis in these cells [87, 121]. Cell migration and proliferation have also been reported to be facilitated by MSCs-EVs [121, 145].

Chondrocyte migration and proliferation are two critical cartilage health mechanisms that are both suppressed in OA. EVs derived from various sources have been shown to increase osteoarthritic chondrocyte proliferation, migration, and viability in a dose-dependent manner [19, 80, 82, 121, 146]. For example, as the EVs dose increases, proliferation occurs sooner; notably, a dose of 10 g of EVs was sufficient to induce chondrocyte migration. Moreover, some studies have examined the proteins involved in chondrocyte adhesion, migration, and proliferation that are regulated by EVs produced from MSCs [147, 148]. EVs alter the expression of genes such as fibroblast growth factor (FGF)-2, survivin, and Bcl2/Bax to control cell proliferation or reverse the inhibitory effects of TNF-α and IL-1β on cell migration and proliferation [87, 117, 121, 146]. By enhancing s-GAG synthesis and suppressing NO and MMP-13 production to maintain stromal homeostasis in a TMJ-OA model, hMSC-EVs attenuated the decrease in proliferation and migration. EVs derived from bone marrow stem cells, adipose stem cells, and synovial stem cells also enhance cartilage regeneration in chondrocytes by increasing GAG synthesis and COL II protein expression, and adipose stem cell-derived EVs exert the most significant effect [149]. Furthermore, these EV protein sources influence ECM stability and actin cytoskeletal dynamics, indirectly increasing chondrocyte proliferation and migration. Moreover, by releasing nucleic acids such as miRNAs, MSCs-EVs can control cell proliferation and migration. The uses of several miRNAs to treat OA are summarized in Table 4.

**Table 4:** miRNAs defined in EVs as a working biomolecule for OA therapy

EV Source	miRNA	Selected animals model in vivo	Role	Pathway	Effect	Reference
UMSCs	lncRNA H19	SD Rats	Promote chondrocyte migration and matrix secretion and inhibit cell apoptosis and senescence	miR-29b-3p/FOXO3	Promote sustained cartilage repair	[86]
AD-MSCs	miR-100-5p	C57BL/6 mice	Promote the proliferation of chondrocytes, increase the level of chondrocytes autophagy, enhance matrix synthesis, and reduce the expression of metabolic factors	mTOR signaling pathway	Protect articular cartilage from damage and ameliorate gait abnormality	[78]
S-MSCs	miR-140-5p	SD rats	Increase the proliferation and migration of chondrocytes	Wnt signaling pathway	Successfully prevent OA	[73]
S-MSCs	miR-155-5p	BALB/C mice	Promote the proliferation and migration of chondrocytes and inhibit cell apoptosis	Runx2	Prevent OA	[75]
hBM-MSCs	miR-136-5p	C57BL/6 mice	Promote the migration of chondrocytes and inhibit chondrocytes degeneration	Targets ELF3	Prevent traumatic OA	[150]
MSCs	miR-135b	SD rats	Promote chondrocyte proliferation and inhibit cartilage degradation	Sp1	Promote cartilage repair	[81]
hMSCs	miR-206	C57BL/6 mice	Promote chondrocyte proliferation and inhibit chondrocyte apoptosis	KLF3-AS1/miR-206/GIT1 axis	Attenuate chondrocyte injury	[19]

### Induction of ECM synthesis

Changes in the composition and organization of the ECM are characteristic of OA. Because COL II and proteoglycans are two of the most important components of the ECM of articular cartilage that contribute to the creation of a healthy cartilage matrix, degradation of cartilage ECM proteins leads to cartilage degeneration [38]. MMP-13 and ADAMTS-4 and ADAMTS-5 are able to reduce the levels of COL II and proteoglycans in the OA joint cavity microenvironment. According to recent research, MSC-EVs can reverse ECM degradation by increasing the expression of matrix proteins and other cartilage formation-related genes while decreasing the levels of matrix-degrading enzymes. Tofio-Vian et al. [79] isolated and identified MVs from hAD-MSCs. When OA chondrocytes were treated with MVs, MMP activity and MMP-13 expression were reduced, but COL II expression increased considerably. Woo et al. [80] also discovered that hAD-MSC-sEVs can reduce the expression of MMP-1, MMP-3, MMP-13, and ADAMTS-5 and increase COL II production.

Many studies have recently examined the role of miRNAs in MSC-EVs to control the ECM. Overexpression of miR-92a-3p in BM-MSCs-EVs increased the expression of cartilage formation-related genes such aggrecan, SRY box gene-9 (SOX9), COL9A1, COL2A1, and cartilage oligomeric matrix protein (COMP) while decreasing the expression of COL10A1, Runt-related transcription factor 2 (Runx2), and MMP-13, according to Mao et al. [151]. Protection provided by EVs is beneficial not only because of the microRNAs contained within EVs but also because of EVs proteins. S-MSC-EVs, for example, contain miR-140-5p, which restores ECM secretion by regulating RalA expression, thereby rescuing SOX9 expression [73]. However, the underlying mechanism remains a mystery. As a result, additional research is needed to confirm the aforementioned findings and determine the mechanisms of cartilage matrix catabolism and anabolism.

### Microenvironmental homeostasis

**Fig. 5:**
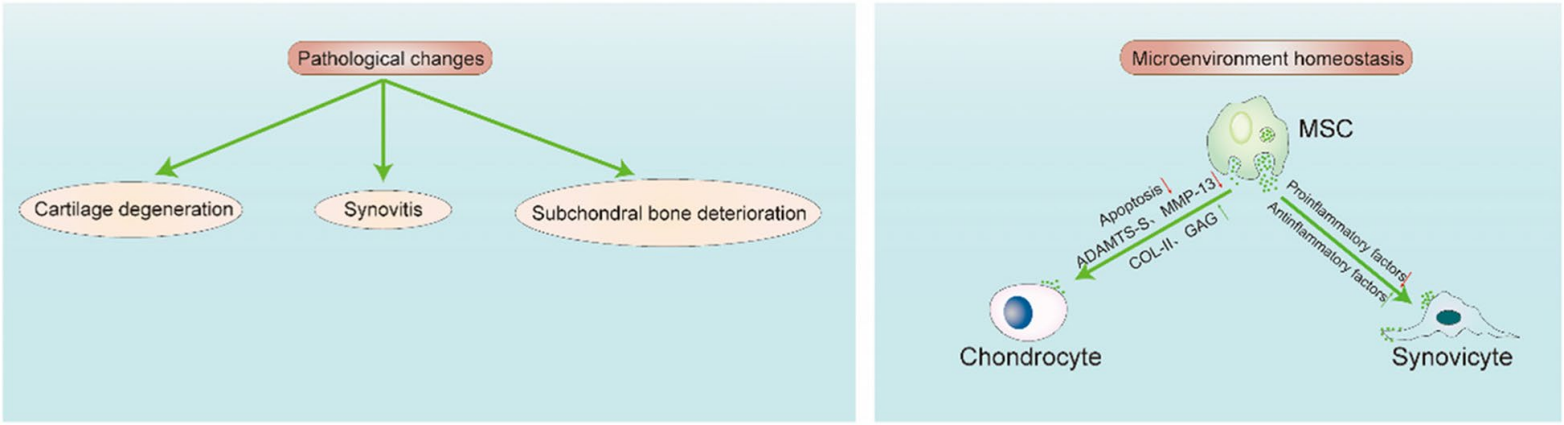
MSCs-EVs target articular chondrocytes and synoviocytes to maintain homeostasis in the articular microenvironment. Cartilage degeneration, synovitis, and subchondral bone degradation are all pathological processes associated with OA. Synovial fluid component contents are altered by MSCs-EVs. Arrows pointing downward indicate downregulation, whereas arrows pointing upward indicate upregulation

MSCs have been proven to have great promise in the treatment of OA in both preclinical and clinical trials. MSCs are involved in tissue homeostasis, free radical scavenging, immunomodulation, and cell proliferation [152, 153]. Substantial data show that the therapeutic effects of MSCs are primarily mediated by paracrine pathways and that MSCs-EVs exert a critical therapeutic effect [21, 73, 121, 154, 155]. Therefore, MSC-EVs maintain homeostasis within the joint microenvironment during OA treatment (Fig. 5).

MSCs-EVs have mostly been used in mice and rats for preclinical therapy of OA and osteochondral abnormalities. MSCs-EVs suppress synovial inflammation, protect subchondral bone, reduce oxidative stress and osteoblast senescence, prevent cartilage and bone from deterioration, correct gait anomalies, stimulate cartilage regeneration, and slow OA development [19, 78, 82, 83, 121]. Table 5 summarizes the in vitro and in vivo data on MSC-EVs.

**Table 5:** Efficacy and molecular mechanisms of EVs derived from MSCs used to treat OA in vivo and in vitro

Source	In vivo	In vitro	References
Human S-MSCs	S-MSC-140-EVs treatment is superior to treatment with SMSC-EVs	SMSC-140-EVs promote chondrocyte proliferation and migration via RalA but do not disrupt ECM secretion	[73]
Human MSCs	MSC-EVs promote cartilage repair better than EVs after lncRNA-KLF3-AS1 knockout	EVs enriched with the lncRNA KLF3-AS1 promote cell proliferation and inhibit apoptosis	[156]
Rat MSCs	TGF-β1-treated EVs promote cartilage repair to a greater extent, and miR-135b inhibitors inhibit the treatment effects	TGF-β1 promotes chondrocyte proliferation through miR-135b enriched in MSC-EVs by regulating Sp1 expression	[81]
Human ESC-MSCs	Protect cartilage and bone from degeneration	Exert similar chondroprotective and anti-inflammatory effects	[21]
Mouse bone marrow MSCs	Prevent cartilage destruction and the process of OA	Maintain the chondrocyte phenotype by increasing COL2A1 synthesis and decreasing ADAMTS-5 expression	[83]
hBM-MSCs	MSC-92a-EVs inhibit the progression of early OA and prevent articular cartilage damage better than MSC-EVs	MSC-92a-EVs increase chondrocyte proliferation and matrix gene expression and target Wnt5A expression	[151]
Human IPFP-MSCs	Protect articular cartilage from damage and improve gait abnormalities; mir-100-5p in the EVs targets the mTOR pathway	Inhibit cell apoptosis and increase matrix synthesis partially by inhibiting mTOR to improve the level of autophagy	[78]
Human MSCs	Not mentioned	Increase the expression of COL2A1 and aggrecan expression and decrease the expression of MMP-13 and Runx2 in OA chondrocytes, attenuate apoptosis in OA articular chondrocytes and lncRNA-KLF3-AS1 targeting of the miR-206/GIT1 axis in EVs	[19]
Rat BM-MSCs	The repair effects on the EV group were significantly better than those on the BMSC and model groups	EVs transfected with siRNA-Piezo1 promote the differentiation of BM-MSCs into cartilage	[157]

Although MSCs-EVs are comparable to MSCs in terms of treating OA and osteochondral abnormalities, they are not the same. Notably, certain drawbacks to using EVs as a clinical translation tool in regenerative medicine have been documented. First, an isolation method that maintain the qualities of EVs in the long term is unavailable. Second, the large numbers of EVs needed for animal investigations and human clinical trials are difficult to attain [158]. Only a few mice can be treated with approximately 1–2 mg (protein content) of EVs generated from a total of approximately 60 million MSCs [145]. MSCs-EVs, on the other hand, are a simpler, safer, more practical, and easier-to-regulate OA therapeutic option than direct cell transplantation.

In conclusion, MSCs-EVs can affect intra-articular cells by controlling cartilage matrix anabolism and catabolism, subsequently enhancing the intra-articular inflammatory milieu, changing intra-articular homeostasis, and curing OA.

## Clinical trial

Due to the advantages of MSC-EVs, many achievements have been reported, and clinical trials have been conducted in other disease fields, including Alzheimer's disease, lung infections, acute respiratory distress syndrome (ARDS), COVID-19, dry eye syndrome, etc. MSC-EVs will inevitably facilitate important advancements in the field of medicine in the future. However, research on the use of EVs as a biological alternative treatment is still in its early stages. The therapeutic use of EVs in the treatment of OA is limited by a variety of issues, as described below: 1. In terms of the illness itself, OA has a complicated etiology that may be caused by a number of different factors, necessitating additional research. 2. From the perspective of EVs, this industry still has certain bottleneck issues: ①for extensive pharmaceutical uses, EV isolation and purification techniques, yield, and purity have not been standardized; ②exosome composition heterogeneity and preservation are difficult problems for industrialization development and ③targeted cells internalization of EVs alters their chemical composition, making subsequent treatment results unpredictable. ④After EVs enter recipient cells, their subsequent biological distribution, pharmacokinetics and specificity of targeted delivery to the specific organ, as well as the therapeutic mechanism of OA disease, have not been fully elucidated. ⑤The location, duration of residency, and biological effects of EVs injected into the articular cavity on normal cells remain unclear.⑥Finally, experimental support for therapy in large animals is insufficient.

### Conclusions and future perspectives

The whole joint, including the cartilage and subchondral bone, is affected by OA. The microenvironment of OA is complicated, and a complete understanding of this microenvironment will be extremely helpful to treat this disease. Because of their unique roles and properties, EVs may control the microenvironmental changes that coordinate the progression of OA, hence delaying disease progression. Furthermore, EVs contain a large number of proteins, miRNAs, and other bioactive molecules that are important for tissue repair and have a wide range of therapeutic applications in the etiology, diagnosis, and treatment of OA. MSCs-EVs inherit the basic activities of their parental cells, and their therapeutic benefits mediated by immunomodulation, tissue cell repair, and regenerative effects may become a key strategy for the treatment of OA. However, these OA studies are currently focused on animal models. The processes are difficult, time-consuming, and expensive, which are the main roadblocks to their usage. As a result, more clinical trials will be required in the future to validate these findings. Engineered EVs have recently become the focus of scientific research to improve the targeting specificity of EVs and enable more precise control. Engineered EVs outperform natural EVs in terms of their therapeutic potential. The study of EVs in OA, including their role, mode of action, and diagnostic/therapeutic applications, is still in its infancy, and many questions remain unresolved. We propose that natural nanomaterial-EVs will be employed as an effective therapeutic strategy for OA patients in the future as technology advances.

## Data Availability

Not applicable.

## Abbreviations

OA: Osteoarthritis
EVs: Extracellular vesicles
IPFP: Infrapatellar fat pad
ECM: Extracellular matrix
IL: Interleukin
TNF-α: Tumor necrosis factor-α
MMP: Matrix metalloproteinases
ACAN: Aggrecan
GAG: Glycosaminoglycan
M1: Proinflammatory macrophages
M2: Anti-inflammatory macrophages
NO: Nitric oxide
PGE2: Prostaglandin E2
CCL5: Chemokine (C–C Motif) Ligand 5
ROS: Reactive oxygen species
COL II: Collagen II
ADAM: A disintegrin and metalloprotease
ADAMTS: ADAM with thrombospondin motifs
Exos: Exosomes
ApoEVs: Apoptotic cell-derived EVs
MVs: Microvesicles
MVBs: Multivesicular bodies
ILVs: Intraluminal vesicles
ESEs: Sorted endosomes
LSEs: Late sorted endosomes
Ago-2: Argonaut-2
ISEV: The International Society for Extracellular Vesicles
ELISA: Enzyme-linked immunosorbent assay
EM: Electron microscopy
AFM: Atomic force microscopy
NTA: Nanoparticle tracking analysis
TRPS: Tunable resistive pulse sensing
DLS: Dynamic light scattering
FC: Flow cytometry
SEA: Single EVs analysis approach
MSCs: Mesenchymal stem cells
S-MSC-EVs: Synovial mesenchymal stem cell-derived EVs
AD-MSC-EVs: Adipose-derived mesenchymal stem cell-derived EVs
BM-MSC-EVs: Bone marrow mesenchymal stem cell-derived EVs
hUMSC-EVs: Human umbilical cord mesenchymal stem cell-derived EVs
S-MSCs-Exos: S-MSCs-Exosomes
Wnt: Wingless / integrated
HMGB: High mobility group protein
3'UTR: 3' untranslated region
MSCs: IPFP-ExosIPFP MSCs-derived Exos
mTOR: Mammalian target of rapamycin
hAD-MSCs: Human AD-MSCs
hAD-MSCs-sEVs: hAD-MSCs-derived small EVs
LFJ OA: Lumbar facet joint OA
CGRP: Calcitonin gene related peptide
RANKL: Receptor activator of nuclear factor-κ B ligand
RANK: Receptor activator of nuclear factor-κ B
TRAF6: Tumor necrosis factor receptor-associated factor 6
iNOS: Inducible nitric oxide synthase
JAK2: Janus kinase 2
KGN: Kartogenin
PTEN: Phosphatase and tensin homolog
Akt: Protein kinase B
lncRNA: Long noncoding RNA
FOXO3: Forkhead box O3
3D: Three-dimensional
2D: Two-dimensional
LAMP-2B: Lysosome-associated membrane protein 2
CAP: Chondrocyte affinity peptide
SF-MSC: Synovial fluid mesenchymal stem cell
NF-kB: Nuclear factor kappa B
IFN-γ: Interferon-γ
TGF: Transforming growth factor
Arg-1: Arginase-1
FGF: Fibroblast growth factor
SOX9: SRY box gene-9
COMP: Cartilage oligomeric matrix protein
Runx2: Runt-related transcription factor 2
